# Letter to the Editor: “Multi-omic profiling reveals age-specific blood biomarkers and aging-driven B cell remodeling in osteoarthritis”

**DOI:** 10.1097/JS9.0000000000003356

**Published:** 2025-09-08

**Authors:** Kun Zhang, Fan Yang

**Affiliations:** a Department of Trauma Orthop, Shenzhen Longhua District People’s Hospital, Shenzhen, Guangdong Province, China; b Department of General Practice, Shenzhen Longhua District People’s Hospital, Shenzhen, Guangdong Province, China

*Dear Editor,*

We read with great interest the recent study on age-related immune dysregulation in osteoarthritis (OA) and the identification of peripheral blood biomarkers through integrated transcriptomic analysis, machine learning, and single-cell RNA sequencing^[1]^. By combining multi-tissue datasets, diverse machine learning approaches, and experimental validation, the authors addressed an important gap in non-invasive OA diagnostics, particularly relevant for older patient populations, a contribution that deserves recognition. While this work offers valuable insights, we believe several points warrant further discussion. This letter to the editor adheres to the TITAN Guidelines 2025 regarding authorship declarations and the responsible use of AI^[2]^.

First, the transcriptomic datasets were obtained from public sources and included four OA-affected joint components. However, variability in sample collection, processing protocols, and patient characteristics could introduce batch effects. Although differential expression and WGCNA analyses were performed, the manuscript would be more convincing if it provided a detailed description of batch-effect correction and confirmed the robustness of identified biomarkers in independent cohorts. Moreover, only one dataset per joint component was used. Other OA datasets are available in the GEO database, such as GSE55457, GSE114007, and GSE51588, and integrating similar datasets could further enhance model robustness and result reliability^[3,4]^.

Second, the peripheral blood biomarker panel (MAPK1, MAP3K8, ING1, LDLR, and NUP153) demonstrated excellent diagnostic performance (AUC = 0.966). Nevertheless, the case–control design and relatively small validation cohort may limit generalizability, especially for early-stage disease or patients with comorbid inflammatory conditions. The exceptionally high AUC in the training set, without external validation, raises the possibility of model overfitting. Future testing in independent external cohorts, ideally across diverse ethnicities and community populations, would help confirm its clinical applicability^[5]^.

Third, the age-stratified analysis revealed intriguing differences in biomarker expression and B-cell phenotypes between older and younger OA patients. While these findings are compelling, the cross-sectional nature of the study precludes establishing temporal or causal relationships. Longitudinal studies tracking disease progression and immune cell remodeling during aging would clarify whether the observed B-cell changes are drivers or consequences of OA pathology.

Finally, the single-cell RNA sequencing analysis elegantly mapped B-cell differentiation trajectories within OA-affected joints, identifying age-enriched activated B-cell subsets associated with chondrocyte injury. Nonetheless, functional studies in vivo or in organoid models would provide stronger evidence for a mechanistic link between age-related B-cell changes and joint degeneration.

In summary, this study advances our understanding of age-specific immune mechanisms in OA and highlights promising blood-based biomarkers. We believe that addressing cohort diversity, incorporating longitudinal validation, and strengthening mechanistic evidence will be important steps toward translating these findings into precise diagnostic and therapeutic strategies.

## Data Availability

Not applicable.
